# Leptin levels are reduced by intravenous ghrelin administration and correlated with cue-induced alcohol craving

**DOI:** 10.1038/tp.2015.140

**Published:** 2015-09-29

**Authors:** C L Haass-Koffler, E G Aoun, R M Swift, S M de la Monte, G A Kenna, L Leggio

**Affiliations:** 1 Section on Clinical Psychoneuroendocrinology and Neuropsychopharmacology, National Institute on Alcohol Abuse and Alcoholism and National Institute on Drug Abuse, National Institutes of Health, Bethesda, MD, USA; 2 Center for Alcohol and Addiction Studies, Department of Behavioral and Social Sciences, Brown University, Providence, RI, USA; 3 Department of Psychiatry and Human Behavior, Brown University, Providence, RI, USA; 4 Center for Alcohol and Addiction Studies, Department of Psychiatry and Human Behavior, Brown University, Providence, RI, USA; 5 Veterans Affairs Medical Center, Providence, RI, USA; 6 Departments of Pathology, Neurology, and Neurosurgery, Rhode Island Hospital and the Warren Alpert Medical School of Brown University, Providence, RI, USA

## Abstract

Increasing evidence supports the role of appetite-regulating pathways, including ghrelin and leptin, in alcoholism. This study tested the hypothesis that intravenous exogenous ghrelin administration acutely decreases endogenous serum leptin levels, and that changes in leptin levels negatively correlate with alcohol craving. This was a double-blind, placebo-controlled human laboratory study. Non-treatment-seeking, alcohol-dependent, heavy drinkers (*n*=45) were randomized to receive intravenous ghrelin or placebo, followed by a cue-reactivity procedure, during which participants were exposed to neutral (juice) and alcohol trial cues. There was a main effect for intravenous ghrelin administration, compared with placebo, in reducing serum leptin levels (*P*<0.01). *Post hoc* analysis showed significant differences in serum leptin levels at the alcohol trial (*P*<0.05) that persisted at the end of the experiment (*P*<0.05). By contrast, there were no significant differences in serum leptin levels at the juice trial (*P*=not significant (NS)). The change of serum leptin level at the alcohol trial correlated with the increase in alcohol urge (*P*<0.05), whereas urge to drink juice was not correlated with the leptin change at the juice trial (*P*=NS). These findings provide preliminary evidence of ghrelin–leptin cross-talk in alcoholic individuals and suggest that their relationship may have a role in alcohol craving.

## Introduction

Alcohol dependence (AD) is a leading cause of preventable morbidity and mortality worldwide.^1, 2^ Medications currently approved by the Food and Drug Administration to treat AD exhibit limited efficacy.^3^ As such, identifying neurobiological pathways involved in the development and maintenance of AD may lead to novel pharmacological targets for AD treatment.^4^

Increasing evidence supports the role of appetite-regulating pathways in addiction.^5, 6^ In particular, ghrelin, a 28-aa (amino acid) residue peptide produced primarily by the stomach, has a key role in increasing appetite, food intake and reward.^7^ Ghrelin was also found to have a role in alcohol reward, alcohol consumption and other alcohol-seeking behaviors in rodents (for review, see refs 5, 8). A longitudinal clinical study revealed a positive correlation between blood ghrelin levels and both alcohol craving and risk of relapse in AD patients.^9^ More recent clinical research indicated that intravenous administration of exogenous ghrelin acutely increased cue-induced alcohol craving in heavy-drinking AD individuals.^10^

Leptin is a feeding-related 146-aa peptide produced by adipose tissue that may also have a role in AD. Serum leptin levels were found to be elevated in AD individuals and associated with increased craving for alcohol.^11, 12^ Moreover, in AD patients, treatment with naltrexone and/or acamprosate was associated with lower leptin levels, suggesting leptin may serve as a possible biomarker for medication response.^13^ Two other peripherally secreted adipose hormones are resistin, a 108-aa protein produced in the stromal vascular fraction of adipose tissue and peripheral blood monocytes^14^ and visfatin, a 491-aa peptide produced by the visceral adipose tissue.^15^ Leptin, resistin and visfatin may have important roles in metabolism and energy balance in several endocrine disorders (for review, see ref. 16). Although the exact physiologic function of resistin in humans is still unknown,^17^ one study reported alteration of resistin levels during alcohol withdrawal in AD individuals.^18^ No clinical findings on visfatin in AD have been reported.

Leptin and ghrelin have inverse appetitive effects; leptin decreases appetite, food intake and reward, whereas ghrelin exhibits opposite roles on these behaviors.^19, 20^ An interaction (‘cross-talk’) between ghrelin and leptin in regulating appetite and reward signaling has been described.^20^ Although both peptides act as afferent signals to the hypothalamus,^21^ leptin and ghrelin receptors are also expressed in motivation reward brain regions linked to food-related behaviors such as ventral tegmental area and substantia nigra that signal via dopaminergic neurons to cortical and limbic region involved in motivational response to both food^22,23^ and drugs.^24, 25^ Consistent with the opposite orexigenic and anorectic effects of ghrelin and leptin, electrophysiological data have shown that treatment of neurons with leptin or ghrelin revealed antagonism between the effect of these two hormones on the neural activity of the arcuate nucleus,^20^ a hypothalamic area with a leaky blood–brain barrier that may favor larger molecules trafficking.^26^

The present study was aimed at investigating the relationship between ghrelin and leptin in alcohol craving by analyzing these serum levels in a human laboratory placebo-controlled study with heavy-drinking AD individuals receiving intravenous ghrelin.^10^ Consistent with the opposite physiological roles of ghrelin vs leptin,^19, 20^ our hypothesis was that intravenous exogenous ghrelin administration acutely decreases endogenous serum leptin levels, and that changes in leptin levels negatively correlate with alcohol craving. Finally, the potential effects of intravenous ghrelin in resistin and visfatin levels were explored.

## Materials and methods

### Setting

This study was conducted at the Brown University Center for Alcohol and Addiction Studies, Providence, RI, USA. The study was approved by the Brown University Institutional Review Board. The use of synthetic human ghrelin was approved under the Food and Drug Administration Investigational New Drug (IND #109,242). Participants signed an informed consent before participation.

### Summary of the parent study

The parent study^10^ was a three-group between-subject, double-blind, placebo-controlled randomized proof-of-concept human laboratory study with 45 non-treatment-seeking heavy-drinking AD individuals (ClinicalTrial.gov: NCT01190085). In brief, individuals were enrolled accordingly to the DSM-IV diagnosis of alcohol dependence; heavy drinking was defined as consuming on average ⩾4 standard drinks per day for women or ⩾5 standard drinks per day for men, during the 90-day period before screening, as assessed by the Timeline Follow-Back. A breath alcohol concentration of 0.00 and being fasting was required on the day of the human laboratory experiment. An intravenous cannula was placed, and a fixed light breakfast of ~700 kJ (~62% carbohydrate, 13% protein and 25% fat) was served. Participants then received an ~10-min administration of intravenous ghrelin at a dose of either 3-μ kg^−1^, 1-μ kg^−1^ or 0-μ kg^−1^ (placebo). Following the intravenous infusion, participants were exposed to a neutral (juice) cues trial and an alcohol cues trial. At the end of each trial, urge was assessed by using either an Alcohol Visual Analogue Scale or a Juice Visual Analogue Scale, both rated on an 11-point anchored Likert-type scales.^27^ Then, another neutral and alcohol cues trial was conducted. Blood samples for hormones were collected at six time points during the experiment, that is, at baseline (before the infusion), immediately following each of the four trials, and finally before discharge (48 min following the infusion).

The main findings of the parent study were a significant effect of ghrelin 3-μ kg^−1^ vs placebo on alcohol craving (*P*<0.05) but not on juice craving (*P*=not significant (NS)). A significant positive correlation between post-infusion blood ghrelin concentration and the increase in urge to drink alcohol was also noted (*P*<0.05).^10^

### Blood samples analysis

The intravenous ghrelin/placebo infusion was a 10-min bolus that took place from –10 min to 0 min. Blood sample time points were at baseline (−15 min), after the first juice trial (+6 min), the first alcohol trial (+17 min), the second juice trial (+23 min), the second alcohol trial (+29 min) and post experiment (+48 min). Blood samples were centrifuged and stored at −80 °C. Leptin, resistin and visfatin levels were determined using a fluorescent bead-based Bio-Plex assay (Bio-Rad, Hercules, CA, USA). Results were expressed as pg ml^−1^.

### Data analytic strategy

Considering there were no significant differences between the two ghrelin doses in the main study,^10^ for this analysis we collapsed the two intravenous ghrelin groups (1-μ kg^−1^ and 3-μ kg^−1^) and compared them with placebo.

Analysis was limited to blood samples collected at baseline (−15 min), during the second juice and alcohol trials (+23 and +29 min) and before discharge (+48 min). In fact, ghrelin’s pharmacokinetics profile predicts that its levels would be positive and relatively constant during this time period, as ghrelin maximum concentration is reached ~10 min following intravenous administration and its half-life (*t*_1/2_) can be as long as 47 min for a bolus injection.^28^ The previous blood samples were collected around *t*_max_ (+6 and +17 min; first juice and alcohol trial, respectively), and as such, would have been too early to detect a possible effect of intravenous ghrelin administration on the endogenous levels of the adipose hormones here analyzed. In addition, previous human laboratory studies using cue-reactivity procedures indicate that elicited craving response is stronger when cue exposure is repeated (that is, during the second trial).^29^ Of note, in the parent study, elicited craving was more pronounced during the second trial.^10^

Serum hormones levels were normalized^30^ to reduce individual and gender-related^31^ variability, evaluated as change from baseline^13^ and expressed as means and standard errors (s.e.m.). Distributional characteristics of outcome measures were examined to evaluate similarity to the normal distribution. Although hormones levels had a slight larger skewness and kurtosis, outliers were taken into account as previously suggested.^32^ Consistent with previous leptin-related literature in the alcoholism field,^11, 13^ hormone/body mass index (BMI) ratio was used as the primary way to express hormones levels. In addition, hormone serum levels were analyzed using BMI as covariate in the model.^33^ BMI was taken into account considering that all hormones investigated here are secreted by adipocytes and that BMI is a measure of adipocyte secreting activity.^16^ The statistical method used was repeated measures analysis of covariance, followed by an unpaired *t*-test to detect the differences in serum hormone levels among groups at specific time points. All pairwise comparisons were covariate adjusted and corrected with a Bonferroni correction (*P*_corrected_). The percent change from baseline was used to evaluate potential correlations with craving scores (Alcohol Visual Analogue Scale or Juice Visual Analogue Scale) via regression analysis using Pearson’s correlation coefficient; this latter analysis was only conducted with the hormones for which a significant intravenous ghrelin effect was detected. All statistical tests were two-sided, and statistical significance was accepted if a *P*-value <0.05 was obtained. SPSS (v.22; Armonk, NY, USA) was used to conduct the analysis and GraphPad Prism (v.5) was used to generate figures (La Jolla, CA, USA).

## Results

### Description of the sample

Out of the 45 participants in the parent study, data for this analysis were available for 43 subjects. Demographics and baseline characteristics are outlined in the parent study.^10^ There were no significant differences in the demographics and baseline characteristics, including basal ghrelin levels between groups in the main sample of the parent study and the sample of this analysis.

**Figure 1:**
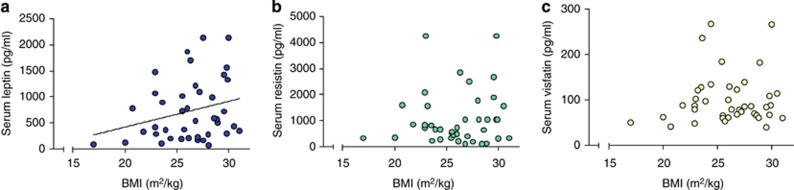
Relationship between baseline adipose hormones levels and body mass index (BMI). There was a trend toward significant positive correlation between baseline serum (**a**) leptin (dark blue) levels and BMI (*r*_41_=0.265; *P*=0.094); (**b**) resistin (green) and (**c**) visfatin (yellow) levels did not correlate with BMI (*P*=NS). NS, not significant.

Consistent with previous studies,^11, 12^ we found a correlation, albeit on a trend level, between BMI and baseline serum leptin levels (*r*_41_=0.265; *P*=0.094; Figure 1a), whereas serum resistin and visfatin levels were not correlated with BMI (*P*=NS; Figure 1b and Figure 1c).

### Changes in endogenous leptin level after ghrelin intravenous infusion

**Figure 2:**
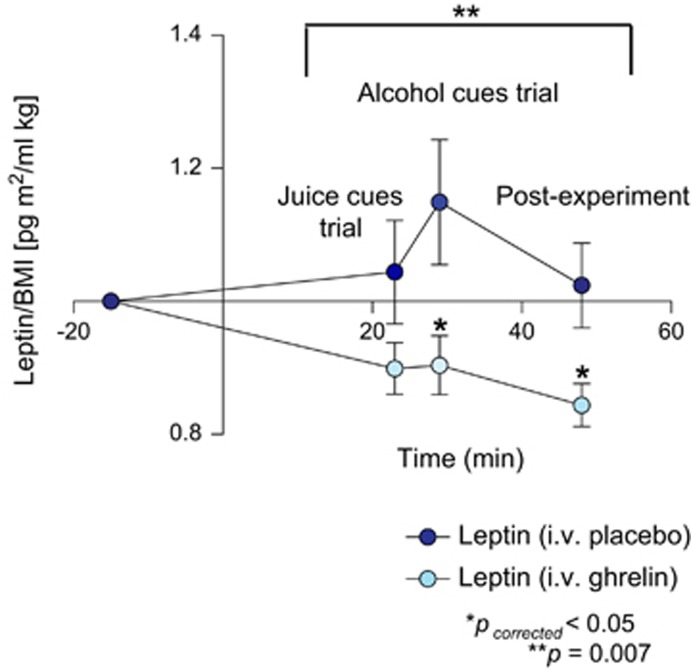
Changes in endogenous serum leptin levels expressed as leptin/BMI ratio after intravenous (i.v.) infusion of exogenous ghrelin (light blue) vs placebo (dark blue). The i.v. ghrelin/placebo infusion was a 10-min bolus that took place from –10 min to 0 min. Leptin time points are at baseline (−15 min), at the juice trial (+23 min), at the alcohol trial (+29 min) and post experiment (+48 min). There was a main effect for i.v. ghrelin administration on serum leptin levels (F_1,38_=8.249, *P*=0.007). *Post hoc* analysis revealed significant differences in leptin levels at the alcohol trial (*t*_38_=2.703, *P*_corrected_=0.031) that persisted at the end of the experiment (*t*_38_=2.720, *P*_corrected_=0.029). No significant differences in leptin levels (*P*=NS) were found during the juice trial (*P*=NS). BMI, body mass index; NS, not significant.

Serum leptin values in the two groups (intravenous ghrelin vs placebo) were normalized and evaluated as change from baseline. There was a main effect for intravenous ghrelin administration in changing serum leptin levels expressed as leptin/BMI ratio (F_1,38_=8.249, *P*=0.007). *Post hoc* analysis revealed significant differences in reducing leptin levels at the alcohol trial (*t*_38_=2.703, *P*_corrected_=0.031) that persisted at the end of the experiment (*t*_38_=2.720, *P*_corrected_=0.029; Figure 2).

These results were confirmed when leptin was expressed as serum level with the BMI used as covariate. Specifically, there was a main effect for intravenous ghrelin administration in reducing serum leptin levels (F_1,37_=7.442, *P*=0.010). *Post hoc* analysis showed a trend toward a significant difference in reducing leptin levels at the alcohol trial (*t*_38_=2.468, *P*_corrected_=0.055) that became significant at the end of the experiment (*t*_38_=2.827, *P*_corrected_=0.022).

By contrast, no significant differences in leptin levels were noted during the juice trial using either leptin/BMI ratio (Figure 2) or serum leptin with BMI as covariate (*P*=NS). Finally, there was no significant main effect on time or medication by time interaction for this outcome (*P*=NS).

### Correlation between serum leptin levels and urges to drink alcohol or juice

**Figure 3:**
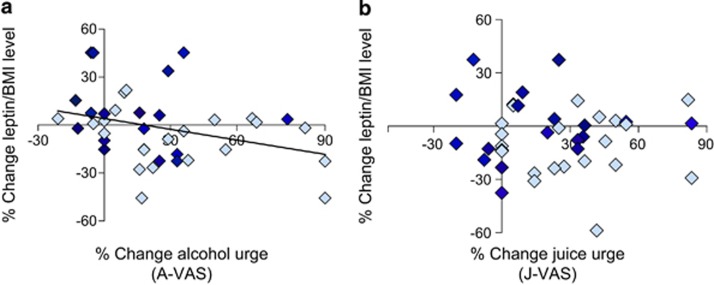
Relationship between serum leptin levels expressed as leptin/BMI ratio after intravenous (i.v.) infusion of exogenous ghrelin (light blue) vs placebo (dark blue) and urge to drink alcohol or juice, measured by the Alcohol Visual Analogue Scale (A-VAS) and the Juice Visual Analogue Scale (J-VAS), respectively. (**a**) Leptin changes were significantly and negatively correlated with the increase in alcohol urge (*r*_40_=−0.342, *P*=0.031) during the alcohol trial (+29 min), (**b**) but not during the juice trial (*P*=NS). BMI, body mass index; NS, not significant.

There was a significant negative correlation between the percent change from baseline in leptin level expressed as leptin/BMI ratio and the percent change in the alcohol urge scale (Alcohol Visual Analogue Scale) during the alcohol trial (*r*_40_=−0.328, *P*=0.039; Figure 3a). We then separately analyzed the regression for each group (intravenous ghrelin or placebo), which confirmed a correlation with alcohol urge (albeit nonsignificant owing to the small underpowered sub-sample) in the same negative direction in both groups, with a stronger effect in the ghrelin group (data not shown). These results were also confirmed when leptin was expressed as serum leptin with BMI as covariate (*r*_40_=−0.342, *P*=0.031). By contrast, there were no significant correlations between the change in leptin and the juice urge scale (Juice Visual Analogue Scale) during the juice trial, expressing leptin either as leptin/BMI ratio (Figure 3b) or leptin level with BMI as covariate (*P*=NS).

### Changes in endogenous resistin and visfatin levels after intravenous ghrelin infusion

Serum resistin and visfatin values were normalized on the same scale and evaluated as change from baseline, as described before for leptin. There were no significant main effects for intravenous ghrelin on resistin/BMI or visfatin/BMI ratios (*P*=NS). These results were confirmed when the hormones were expressed as serum levels with BMI as covariate (*P*=NS). There was no significant main effect on time or medication by time interaction for these outcomes (*P*=NS).

## Discussion

Consistent with our hypotheses, the main findings of this analysis were that intravenous ghrelin administration significantly decreased serum leptin levels, and ghrelin-induced changes in leptin levels significantly negatively correlated with increased alcohol craving in AD heavy drinkers. We believe this is the first study in which the effects of exogenous intravenous ghrelin administration on endogenous serum leptin levels were assessed in the context of alcohol craving studied in AD individuals. This study provides preliminary evidence supporting a possible ghrelin and leptin relationship (‘cross-talk’) in AD individuals and suggesting that their interaction may have a role in alcohol craving.

These results hold important clinical value because craving may be associated with relapse and has been proposed as a clinically relevant endophenotype able to predict alcohol-related outcomes.^34^ Notably, cue reactivity has demonstrated utility in eliciting urge to drink in AD individuals^29^ and medications that reduce alcohol consumption also reduce alcohol craving in cue reactivity human laboratory studies.^35^

Our parent study^10^ showed that intravenous ghrelin administration resulted in an acute increase in cue-induced urge to drink alcohol; during the experiment, there was also a significant positive correlation between the post-infusion serum ghrelin levels and the increase in urge to drink alcohol. The findings of the present study are consistent with those of the parent study, and also add important information. While the significant positive correlation between post-infusion blood ghrelin levels and the ghrelin-induced increase in urge to drink alcohol reported in the parent study was expected, here we report that a different hormone, leptin, significantly decreased after intravenous ghrelin infusion and its decrease significantly correlated with the change in urge to drink alcohol. As such, leptin may represent a biomarker of ghrelin’s effects on alcohol craving in AD individuals. This is relevant, as leptin acts centrally via a negative feedback loop regulating body weight^36^ and counteracting ghrelin response.^37^ Three additional observations provide support suggesting a possible specific role of leptin as a pharmacodynamic biomarker of ghrelin’s effects in the urge to drink alcohol: (1) the correlation between ghrelin-induced changes in leptin and craving was specific for the urge to drink alcohol, as similar results were not found for the urge to drink juice; (2) the correlation was specific for leptin, as no similar results were found for resistin or visfatin; and (3) although this study did find an effect of intravenous ghrelin on leptin levels in a population of AD patients, it is important to note that in a previous small study with healthy controls, intravenous ghrelin did not change leptin levels.^38^ It is possible to speculate that ghrelin’s effects on alcohol craving might be mediated, at least in part, via its effects in specifically reducing leptin levels, which in turn may affect alcohol-seeking behaviors or that leptin may have a protective effect, at least under some conditions, on alcohol craving and drinking. Although this study does not allow us to directly test this hypothesis, we note that putative protective effects of leptin have been reported, as increased leptin serum levels facilitate learning and memory performance in preclinical studies,^39^ and have a neuroprotective role in ischemic brain injury.^40^ Translational studies, however, are needed to fully address the possible cross-talk between ghrelin and leptin in alcohol craving and drinking.

The positive correlation between serum ghrelin levels and craving (parent study^10^) and the negative correlation between serum leptin levels and craving (present study), together, are consistent with the opposite physiological effects of these two hormones,^19, 20^ as well as with the observation that intracerebral administration of ghrelin reversed the effects of leptin in rodents.^41^ In addition, our findings are consistent with a recent study where, after alcohol abstinence, AD patients had opposite changes in the endogenous levels of these two peptides, that is, elevated ghrelin and reduced leptin levels.^42^

Some studies suggest a role of leptin itself in alcohol craving,^11^ withdrawal-induced craving,^12^ including as a possible biomarker to assess medication response^13^ in AD individuals. However, while our findings indicate a negative correlation between leptin and craving, others reported a positive correlation.^13^ This inconsistency may be owing to several factors, for example, differences in the nutritional status of the subjects across studies, different fasting conditions, potential differences in alcohol drinking and severity of dependence, among the others. The most important difference, however, is that in the study by Kiefer *et al.*,^13^ leptin was measured in a placebo-controlled study testing naltrexone and acamprosate, whereas here we studied leptin in a placebo-controlled study testing intravenous ghrelin. In that regard, the present study addresses primarily the potential ghrelin–leptin link in alcohol craving, rather than a role of leptin itself in craving.

From a neurobiological prospective, leptin receptors are expressed in the brain in the satiety centers of the hypothalamus^43^ and peripherally in the adipocytes^44^ indicating the autocrine and paracrine role of leptin in energy balance (for review, see ref. 45). In the brain, they are also co-expressed with ghrelin receptors mostly in the hypothalamus, and less extent in the other parts of the brain.^46^ Interestingly, at the peripheral level, ghrelin directly enhances *in vitro* adipocyte leptin release,^47^ and *in vivo* the expression of ghrelin receptors increases with adipogenesis.^48^ Taken together, these findings may support the possibility of ‘adiposity signals’ that communicate directly to the brain,^37^ where ghrelin orexigenic effects antagonize leptin action.^49^

Fat storage has been identified as the most important variable that determines serum leptin levels^50^ and the positive relationship between leptin and BMI found in our sample is consistent with what previously described.^51^ The lack of similar relationship between BMI and either resistin or visfastin is also consistent with previous literature. In fact, the lack of correlation between resistin and human adiposity, weight and BMI has been already reported (for review, see ref. 17), probably owing to the low resistin messenger RNA expression in human adipocytes^52^ compared to rodents.^53^ Similarly, even if secreted by adipocytes, visfatin is highly expressed in lean individuals.^54^ Although the different relationship with BMI shown by leptin vs resistin or visfastin is in line with the previous published reports, this study allowed us for controlling very carefully for adiposity and BMI as potentially confounders. In fact, the positive findings on leptin were similar when leptin was expressed as either leptin/BMI ratio or leptin levels with BMI as covariate in the model. In addition, ghrelin infusion was dosed by weight,^10^ rather than using a fixed bolus dose as in the other studies (for review, see ref. 55), thus ensuring that the changes in endogenous leptin levels, driven by exogenous intravenous ghrelin, were controlled by body fat mass. Finally, the effect of intravenous ghrelin infusion was selective for leptin only and evident at the alcohol trial.

This research has a number of strengths, in fact this study (a) was the first to study leptin signaling after an intravenous ghrelin challenge in AD individuals; (b) included a well-validated procedure (that is, cue reactivity); (c) the leptin–ghrelin relationship was investigated in a strict and well-controlled environment, thus allowing us to measure in real time cue-elicited craving and control carefully for several possible confounders (for example, recent alcohol and food intake); (d) as mentioned before, intravenous ghrelin dose was calculated on the basis of weight, an important factor for an analysis like this focused on the ghrelin’s effects on adipose hormones; (e) normalization of serum leptin at baseline and the percent change from baseline allowed us for accounting for individual variations in circulating leptin levels, including sex-related differences; and (f) the consistency of intravenous ghrelin effect on leptin levels was shown using two separate computational methods. Limitations of the study include: (a) the small sample; (b) the short duration of intravenous ghrelin administration; and (c) the fact that obviously this study does not answer the complementary question of how endogenous ghrelin may vary in AD individuals after an acute exogenous leptin challenge. It is also important to note that other gut-brain peptides regulating appetitive hormones also have a role in alcoholism (for review, see ref. 56), but were not included in this analysis. An additional consideration is that juice and alcohol cues were presented in a fixed order, which may be seen as a limitation. However, the use of a fixed order was done to allow for the most conservative assessment of alcohol cue reactivity^27, 29, 57^ as previous studies reported a general lowering of cue reactivity to any stimulus presented second.^57^

In conclusion, the present study provides preliminary evidence of a ghrelin–leptin cross-talk in AD individuals and that their interaction may have a role in alcohol craving. Future translational research is needed to shed light on the potential role of the interaction of these two pathways in alcohol craving and drinking.
